# Biodiversity conservation: an example of a multidisciplinary approach to marine dispersal

**DOI:** 10.1007/s12210-014-0357-2

**Published:** 2014-12-02

**Authors:** Stefano Aliani, Maristella Berta, Mireno Borghini, Daniel Carlson, Alessandra Conversi, Lorenzo Corgnati, Annalisa Griffa, Marcello Gatimu Magaldi, Carlo Mantovani, Simone Marini, Luca Mazzei, Giuseppe Suaria, Anna Vetrano

**Affiliations:** 1 CNR-ISMAR (Institute of Marine Sciences, National Research Council of Italy), Forte S. Teresa, 19032 Pozzuolo di Lerici, SP Italy; 2 Centre for Marine and Coastal Policy Research, Marine Institute, Plymouth University, Plymouth, PL4 8AA UK

**Keywords:** Biodiversity, Connectivity, Physical–biological coupling, Ocean model and measurements, Marine protected areas

## Abstract

The general aim of this paper is to present a possible multidisciplinary approach to the problem of connectivity among marine protected areas (MPAs) describing some of the mechanisms and vectors that control the dispersal of propagules among spatially distributed marine communities of MPAs in the Southern Adriatic Sea. A joint approach is described that focuses on (a) measurements of surface water current and model data integrated with a dedicated software (LAVA, LAgrangian Variational Analysis), (b) measurements of rafting objects and their evaluation as an alternative way to species dispersal, and (c) a tool to automatically monitor propagules and plankton species in the water column. Studies on the dynamics of water currents demonstrated that the Gargano area has the potential to supply dispersal propagules to the Southern Adriatic both along the Italian coastline and offshore across the basin, thus providing important services to the dispersal processes and the connectivity routes among MPAs. The natural dispersion is however enhanced by floating objects, on which entire marine communities are living and travelling. The number of these objects has greatly increased with the introduction of human litter: in the Adriatic, man-made litter composes nowadays the majority (79 %) of all floating objects, with this corresponding to an almost fourfold increase in the abundance of floating objects since pre-industrial times. Such enhanced dispersion may benefit transmission of propagules from MPAs along biodiversity corridors, but may also enhance the arrival of invasive species. The direct observation of organisms can provide information on the species distribution and mobility. New technology (GUARD-1 system) has been developed to automatically identify spatial or temporal distributions of selected species in the water column by image analysis. The system has so far successfully detected blooms of ctenophores in the water column and is now being tested for identification of other zooplankton groups, such as copepods, as well as marine litter. This low-cost, long-lasting imaging system can be hosted on mobile devices such as drifters, which makes it very suitable for biological dispersal studies.

## Introduction

Preserving biodiversity is the challenge of the future. After having disrupted most habitats in the recent past such that nowadays no pristine environments exist on Earth (Jackson 1997), and ecosystems have been altered by sustained direct interactions with human systems (Ellis et al. 2013), we are now facing the problem of preserving and restoring.

Marine protected areas (MPAs) are one of the most used tools to preserve and recover marine environment. Most of them have been established in the last century based upon a “nice looking” and “low economic impact” basis, especially in the Mediterranean Sea. Their purpose was to protect beautiful places while minimising the economic impact of removing these areas from commercial exploitation.

Nowadays MPAs have reached a more complex function, and the wide array of ecosystem services that they provide is being recognized (Nunes et al. 2014). As knowledge evolves, the concepts of ecosystem services and economic value of biodiversity are expected to drive environmental management (Potts et al. 2014). Managing individual ecosystem services in isolation however ignores the inherent synergies and tradeoffs among them (Halpern et al. 2010) and new approaches include amplifying their connections. Established MPAs are now foreseen as drops on a line along a set of biodiversity corridors, connected by dispersal agents, but also reaching remote areas, thus enhancing biodiversity potential.

Description of connectivity pathways and vectors, and identification of species to and from MPAs are therefore a fundamental part of protection and management, because knowledge of processes and impacts shall sustain 21th century managers in taking decisions.

Most marine animals spend part of their life cycles as plankton, and many marine species feed on plankton for part of, or for their entire life (Hays et al. 2005). Water currents are the major means of transport for planktonic species. Currents transport phytoplankton and zooplankton, medusae, nekton and benthic larvae over distances that are far out of their swimming capabilities. Therefore the knowledge of water dynamics, circulation and transport, and of their drivers, is a necessary prerequisite for understanding marine biodiversity patterns, and a compulsory information for environmental management. The role of water dynamics in the management of MPAs has been generally underestimated when MPAs were originally established (Roff 2005), but it is becoming crucial now that we are moving from MPAs to networks of MPAs, in order to maximise their impact on biodiversity conservation.

Ocean currents can be studied using various types of instruments. For the ocean surface the most effective platforms providing information on velocity and associated transport are drifters and coastal high frequency (HF) radars. Their measurements are in some respect complementary, and they have been used in a number of transport studies (Molcard et al. 2009; Haza et al. 2010). HF radars provide continuous monitoring of surface velocities over extended areas (range up to 100 km), producing velocity maps every 0.25–1 h with spatial resolution of the order of 1–3 km. These velocity fields can be used, for instance, to compute trajectories of particles acting as proxies for eggs, larvae or pollution parcels. Drifters are small floating buoys (radius of the order of 1 m) that follow the currents with good approximation, communicating their position (and other environmental parameters) via satellite at time intervals of the order of a few minutes. They provide direct and precise information on transport, even though they have to be launched ad hoc at times and places of interest.

Optimised strategies include a joint use of both HF radar and drifters, in order to take advantage of both spatial coverage and direct transport information (Berta et al. 2014). The blending of such complementary information through the LAVA algorithm (LAgrangian Variational Analysis) can enhance the knowledge of local transport to which several biological processes are connected (Bi et al. 2011; Franks and Anderson 1992; Zhao and Guo 2011).

Numerical ocean models can be used to perform predictions, test different scenarios and also provide gridded velocity information at depth, where oceanic measurements are usually very scarce. The effects of small, turbulent oceanic features on transport depend on model accuracy. Model forecast capability improves with resolution and in areas where data have been assimilated and used for validation (Olascoaga and Haller 2012; Kuang et al. 2012).

Traditionally, the distribution of organisms has been supposed to depend directly on the length of the larval or planktonic cycle: the longer the period, the longer the transport by currents, and the wider the distribution of organisms (Thorson 1950). Planktonic stages are not the only effective means of dispersal, however (Aliani and Meloni 1999). A series of natural dispersal vectors have been identified at different spatial and temporal scales, but increasingly more are of anthropogenic origin. For example, hitchhiking on floating objects, or transport by ballast waters (Aliani and Molcard 2003; Galil 2000), therefore, the pitfalls of estimating the dispersal potential of organisms solely on the duration of their pelagic larval stage should be considered.

In the last 30–40 years for instance, the massive introduction of man-made debris into the marine environment has enormously increased the opportunity for transport and dispersal of marine fauna into new habitats (Thiel et al. 2003; Barnes and Fraser 2003; Barnes and Milner 2005; Thiel and Gutow 2005). Floating items in fact, are often fouled by a wide range of marine organisms and, according to Barnes (2002), the increasing presence of man-made litter has roughly doubled the propagation of fauna in the subtropics and more than tripled it at high latitudes in comparison to pre-industrial litter-free conditions. Although the relative contribution of man-made litter needs to be considered alongside with other natural or anthropogenic vectors (such as transport on natural objects or on the hulls of ships, or release of ballast water), it is now clear that rafting on plastic debris may facilitate the transport of species across boundaries of water masses that might otherwise be relatively impenetrable (Barnes et al. 2010; Derraik 2002; Gregory 2009). In spite of the growing body of evidence supporting these findings, knowledge on the extent of this process and on its implications for future environmental management is still lacking.

The direct observation of species can provide additional information on their dispersion and biogeographical range. Traditional sampling surveys take repeated samples over the same location, which do not take into account the water mass dynamics. Thus, an autonomous system that can follow the water mass can provide the biological information associated with it. Here we present an autonomous imaging instrument, the GUARD1 system, which has been developed with the aim to autonomously monitor zooplankton (Marini et al. 2013). The GUARD1 device is a suitable tool for autonomous monitoring and automatic recognition of zooplankton from fixed and mobile platforms. Furthermore, its low cost, low volume and low power consumption make it ideal for long-lasting deployments.

The general aim of this paper is to present a possible multidisciplinary approach to the problem of connectivity among MPAs, using as a case study some processes and vectors that control the dispersal of propagules among spatially distributed marine communities of MPAs in the Southern Adriatic Sea. This multidisciplinary perspective is meant to provide a new ecosystem-based vision which can aid in the management of MPAs.

## Methods

Here, the main methodologies used in the paper are introduced and briefly described. They include (a) direct observations of ocean currents using HF radars, (b) numerical setup of dedicated circulation models, (c) LAVA blending techniques that merge information from drifter trajectories and radar (or model) velocity fields, (d) observations of floating debris through dedicated surveys and (e) investigation of plankton abundance using the GUARD1 imaging system.

### HF radar

A dedicated observing system composed of four HF radars complemented by drifters was deployed in the Southern Adriatic Sea during the period from April 2013 to May 2014 in order to study transport pathways and connectivity in the framework of the Italian national projects “Decision support system for a sustainable management of fisheries in South Italy” (SSD-PESCA) and “Ricerca italiana per il mare” (RITMARE), and the European project CoCoNet (toward a coast-to-coast network of MPAs coupled with sea-based wind energy potential). The HF radar network consists of four SeaSonde by CODAR Ocean Sensor (Barrick et al. 2008) with emitting frequencies of 25 MHz and a typical range of coverage of 30 km. The radar system has a resolution of 1.5 km for vector velocity. Accuracy has been tested comparing radial velocities from radar and drifters. Results from eight drifter launches indicate an average root mean square (RMS) difference between radar and drifters of 4.2 cm/sec, while RMS of drifter velocity is 14.2. These values are in the low range of expected differences (Molcard et al. 2009), and they indicate a very satisfactory level of accuracy of the radar system.

**Fig. 1:**
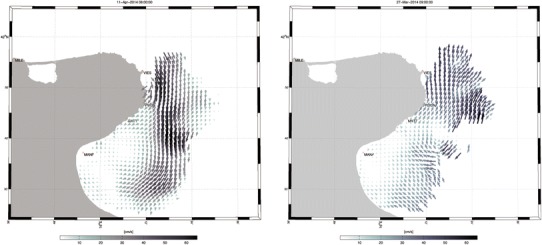
Surface water currents around Gargano area as measured by HF radar. In the* left picture*, a boundary current flowing south along the Italian coast and detaching from the Gargano Cape, and an anticyclonic recirculation in the interior of the Gulf are visible. In the* right picture*, previous typical pattern was disrupted and reversed as function of wind. In particular, *Scirocco* wind from the south can reverse the boundary current, inducing a northern flow

The system was first installed on the northern coast of Gargano, in front of the Tremiti Islands, during the months of April–May 2013 to monitor the effects of the Adriatic boundary currents. Since October 2013 the antennas were moved to the Gulf of Manfredonia (see Fig. 1), where they have been operational until now (http://radarhf.ismar.cnr.it/). The Gulf of Manfredonia and the Gargano are nursery areas for most pelagic fishes (anchovies and sardines) and especially interesting from the environmental point of view. They are also close to some established Adriatic MPAs, like the Tremiti Islands.

### Numerical model

**Fig. 2:**
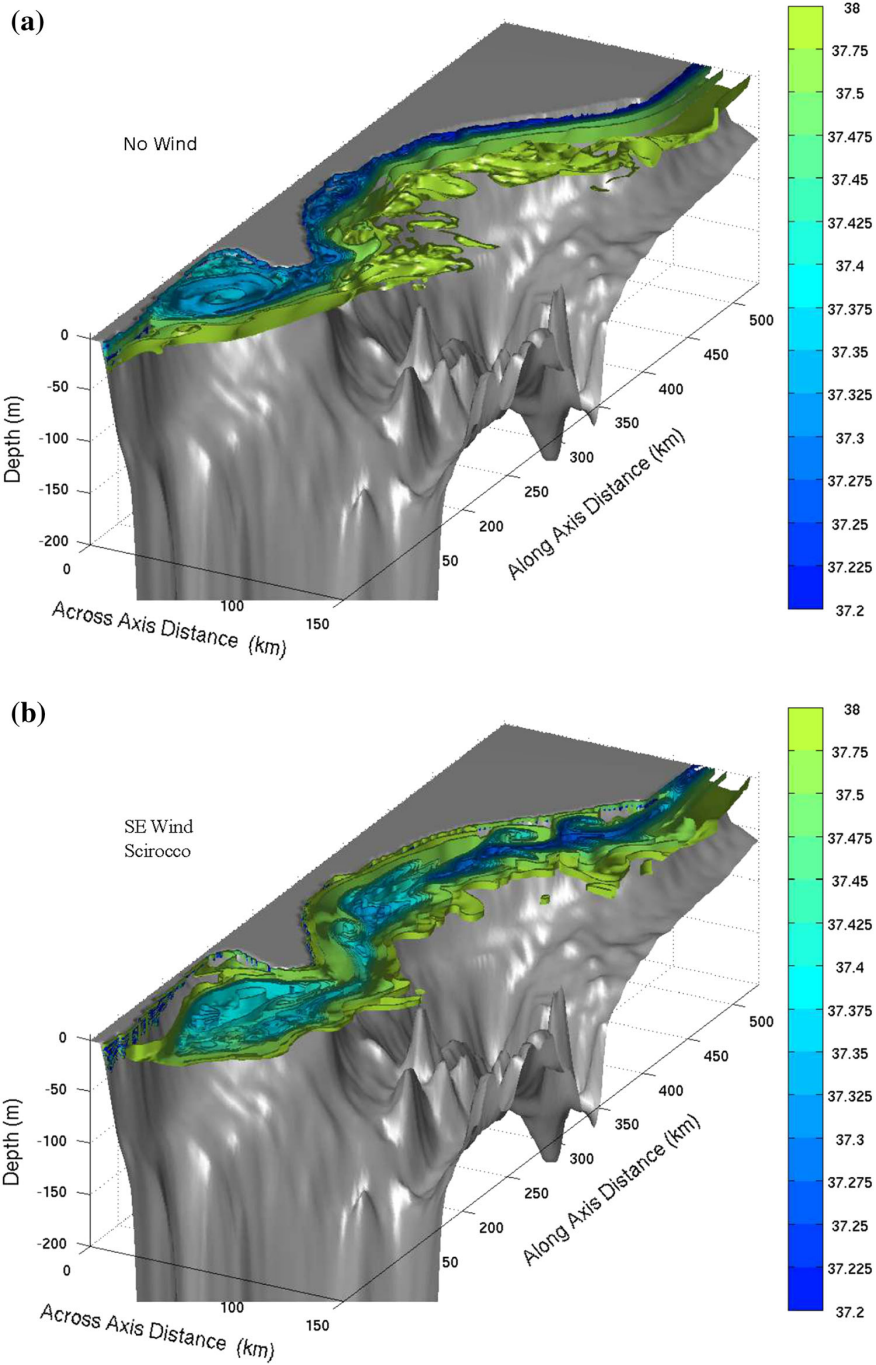
Salinity surfaces in the Gargano area from the 2-km resolution ROMS simulations under different wind conditions modified after Magaldi et al. (2010).** a** Simulation with no winds.** b** Simulation with *Scirocco* winds. Instabilities perpendicular to the coastline are highlighted, which may enhance cross-shelf transport from coastal MPAs close to Gargano toward the open ocean circulation

The Regional Ocean Modeling System (ROMS) is a numerical model that integrates the primitive equations to calculate the three-dimensional velocity, temperature and salinity fields in the ocean. The reader is referred to Shchepetkin and McWilliams (2005) for more information on the different options available for ROMS and for its numerical solvers. ROMS has been configured and validated against observations for the Adriatic Sea as detailed in Magaldi et al. (2010), the numerical grid is centred around the Gargano Promontory; it is 530 km long, 230 km wide and rotated 60° clockwise with respect to true north. To assess the role of winds in forcing water currents around Gargano, different idealized and realistic experiments have been set up (Magaldi et al. 2010). Two idealized experiments are of particular interest to this study (see Fig. 2). In the first idealized experiment, the flow evolves in the absence of winds. The second idealized experiment is run with southeasterly (*Scirocco*) winds, held at a constant value of 7 m s^−1^. Both experiments have a nominal horizontal resolution of 2 km, while the vertical dimension is discretized by 30 stretched terrain-following “*sigma*” layers.

### The LAVA blending method

LAVA is a variational algorithm, which is especially suited for transport studies (Taillandier et al. 2006a). The velocity fields derived from models or from HF radar observations can be further improved by blending them with observations from drifters (small buoys floating along currents). In fact, the deployment of drifters would allow to monitor transport processes that exert control at key phases of nutrient cycles and dispersion of plankton patches. Generally CODE drifters are used since they are designed to closely follow currents within the first meter of the water column. Comparison with current meters shows that errors are within 1–3 cm/s for winds up to 10 m/s (Davis 1985; Poulain et al. 2009). LAVA corrects a first-guess velocity field, coming from a model or radars, by minimizing the distance between observed drifter positions and numerical trajectories advected in the velocity field. The correction is applied along the trajectory and it is spread through a diffusion equation over a range *R* (Derber and Rosati 1989; Weaver and Courtier 2001). The length of *R* is typically scaled with the Rossby radius of deformation characteristic of the targeted area, in order to enhance the drifters’ information associated with the mesoscale.

### Rafting objects

A large-scale survey of floating macro (2–10 cm) and mega (>10 cm) debris was conducted on board R/V Urania during May 2013 across the entire Central and Southern part of the Adriatic Sea. Sightings and samples provided information on the occurrence and distribution of floating debris in the Adriatic basin, as well as an estimation of the potential impacts that floating litter may have on the regional connectivity patterns. A total of 30 visual transects were conducted, covering an overall surveyed length of 277.6 km. Sightings were all performed from the deck during regular navigation. Size, type, perpendicular distance and GPS position of all sighted items were recorded as well as environmental data, boat speed and length of the transects. After being recorded, each debris item was allocated, according to type and origin of material, into two major categories: anthropogenic marine debris (AMD) and natural marine debris (NMD). Then, based on number of sightings, mean densities of AMD and NMD (expressed as items/km^2^) were computed for each surveyed transect using a simplified distance-sampling technique which takes into account also the decrease in sighting effectiveness with increasing distance from the observer (Buckland et al. 2005). Some samples of floating debris were also collected and preserved in alcohol and organisms living on flotsam were identified in the field at the lowest taxonomic level possible. Some pictures of rafting objects were also acquired.

### GUARD1: automatic visual detection system

The GUARD1 device acquires underwater images of objects or organisms from 1 mm to 100 cm of size (e.g., gelatinous zooplankton, fishes, litter). The acquired images are processed on board, where the analysis software recognises and classifies the image content, through pattern recognition algorithms combining computer vision and artificial intelligence methodologies. All the image acquisition parameters are programmable (e.g., ISO, exposure time, focal length, iris aperture) as well as the image acquisition frequency. The system idles in a stand-by status between two consecutive image acquisitions and the computer vision algorithms work on groups of acquired images (not on single images) at scheduled time intervals. This behaviour permits a very long time deployment of the device. The GUARD1 device, installed on fixed or mobile platforms, is particularly suitable for studying gelatinous zooplankton dynamics (horizontal and vertical distribution and migration) as well as for patrolling tasks along selected transects, for example, to detect blooms of jellies and provide early warnings in case of jelly invasion. The instrument can also be used to recover large scale and long-term information, where the monitoring task should rely on long deployment of gliders and expendable drifters and Argo floats. This system is particularly suitable for following organisms and objects along a current, because it can be installed on drifters buoys and can provide visual information on every relevant object encountered. Transmission via radio link of processed data at scheduled intervals will be implemented. The imaging system has so far been tested in the Ligurian Sea, and will in future be deployed in the Southern Adriatic.

Some of these methods, although sometimes used in other seas, are reported here because of their methodological value and because similar future applications are planned in the Adriatic.

## Results

The different approaches proposed in this work provide complementary information on the topic of dispersal: current reconstructions provide information on natural dispersion, floating objects provide an estimate of enhanced (mainly anthropogenic) dispersal, while the automatic recognition method provides direct sightings of zooplankton. The results of these different approaches are presented in this section.

### HF radar and numerical models for understanding natural dispersion

The actual retention properties of the Gulf of Manfredonia and its connection to remote spawning areas have never been measured before, and our data from the HF system (Fig. 1) are expected to shed some light on the hydrodynamical processes underlying these phenomena. Preliminary analyses indicate that the hydrodynamic system in the Gulf is much more complex than previously thought. The expected “typical” behaviour of the currents may be inferred from the numerical simulations without winds as shown in Fig. 2a. It consists of a main boundary current flowing south along the Italian coast and detaching from the Gargano Promontory. In the inner Gulf, a large anticyclonic recirculation forms due to the process of separation behind an obstacle (Schlichting and Gersten 2003), in this case represented by the Gargano. Even without winds, smaller instabilities are present at the outer edge of the current, as shown by the three-dimensional salinity surfaces calculated in the model (Fig. 2a). A similar pattern is shown also by the HF radar results (Fig. 1a), even though the anticyclonic eddy appears smaller and more attached to the coast. Variability is very high, with hourly changes in velocity patterns. This typical pattern changes as a function of wind direction. With *Scirocco* winds (Figs. 1b, 2b), the boundary current reverts, inducing a northern flow. At the same time, edge instabilities may develop and grow, enhancing offshore transport. As a result, complex small-scale structures and submesoscale gyres are also often present in the Gulf interior.

### LAVA blending of radar and model outputs with drifters for improved transport estimates

The LAVA method has been previously used in the Adriatic Sea, blending information from drifters with the outputs of a ROMS model (Taillandier et al. 2008). Results show that the method is very effective, impacting surface current transport especially in terms of export rates from the boundary current toward the interior. Given the restricted set of drifters, though, quantitative testing was limited. Therefore, in the near future, LAVA will be employed in the Adriatic Sea to blend radar and drifter data because several biological phenomena related to MPAs have been observed to be connected to local transport variability (Kršinić and Grbec 2006; Marasović et al. 1991; Vilicic et al. 2013). This new application is encouraged by the previous successful applications of LAVA over modeled currents in different areas of the Mediterranean and China Seas (Taillandier et al. 2006a, b, 2010; Chang et al. 2011), over satellite altimetry-based velocity fields (Berta et al. 2014b) and, very recently, on the HF radar currents dataset in the Toulon area (NW Mediterranean Sea) (Berta et al. 2014).

In the application in the NW Mediterranean Sea, several drifters have been released and a subset of them has been blended with the radar velocities via LAVA. The remaining (control) drifters have been used to assess the effectiveness of the blending in terms of Lagrangian transport estimates.

**Fig. 3:**
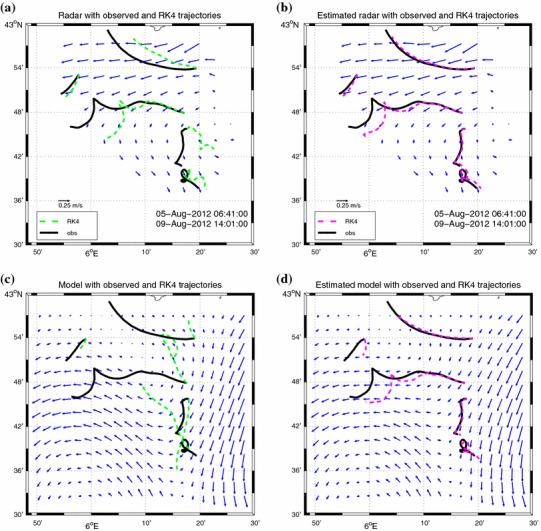
Comparison between observed drifter trajectories (*solid lines*) and simulated trajectories (*dashed lines*) computed from different velocity fields: ** a** original radar velocity; ** b** LAVA-blended radar velocity; **c** original model velocity; ** d** LAVA-blended model velocity. The trajectories are superimposed on the corresponding velocity averaged over the period 5–9 August. Arrows size and period considered in the * lower panels* are as in the upper ones. Modified after Berta et al. (2014)

In Fig. 3 the effects of LAVA on hindcast trajectories are shown both for radar and model. For the radar case (panel a) numerical trajectories (dashed lines) calculated on the measured field are already very similar to the observed drifter trajectories (solid lines). On the contrary, the trajectories calculated over the model velocity field (dashed lines in panel c) are different from the drifters (solid lines) and, in some cases, they diverge moving in opposite directions. When LAVA is applied, the trajectory estimates are largely enhanced. This is true for radar (panel b) but especially for the model case (panel d). When radar currents are used to estimate particle positions, the average uncertainty within 24 h is about 7km. For model currents the error is about 14 km and comparable with the average distance travelled by control drifters (Ullman et al. 2006), i.e., 13 km. When LAVA is used to blend drifter information in the velocity fields provided by the radars or by the model, position uncertainties are diminished to about 1–2 km for both applications.

### Rafting objects and enhanced dispersion

In addition to current dispersal, enhanced dispersal is caused by species, and often entire communities, being transported along currents on floating objects. To evaluate their significance we used visual sightings through the Southern and Central Adriatic. Despite a considerable heterogeneity in debris abundances, floating objects were sighted throughout the entire study area. Man-made objects resulted to be markedly more abundant than natural items across all surveyed locations. A detailed breakdown of debris densities can be found in Suaria and Aliani (2014). We recall here that during our survey, a total of 357 floating items were recorded in the study area, 79 % of which were of anthropogenic origin (i.e., plastic, styrofoam and other man-made objects). Anthropogenic marine debris (AMD) was sighted in 93 % of the 30 transects performed, with abundances ranging from 0 to 104.2 items/km^2^ and a mean total density of 33.6 ± 5.6 items/km^2^ across the entire survey area. On the other hand, natural marine debris (NMD) was found only in 50 % of all transects showing a significantly lower mean density of 8.9 ± 2.2 items/km^2^. With the only exception of the Strait of Otranto, where an extremely high AMD/NMD ratio of 40.3 was found, the mean ratios between anthropogenic and natural debris in the rest of the Adriatic Sea ranged from 2.6 (in the Southeastern Adriatic) to 3.9 (in the Central Adriatic). On average the AMD/NMD ratio across all surveyed locations was 3.8, with this meaning that there has been almost a fourfold increase in the chances for an Adriatic organism to encounter and hitchhike a floating object, if compared to preindustrial litter-free conditions.

**Fig. 4:**
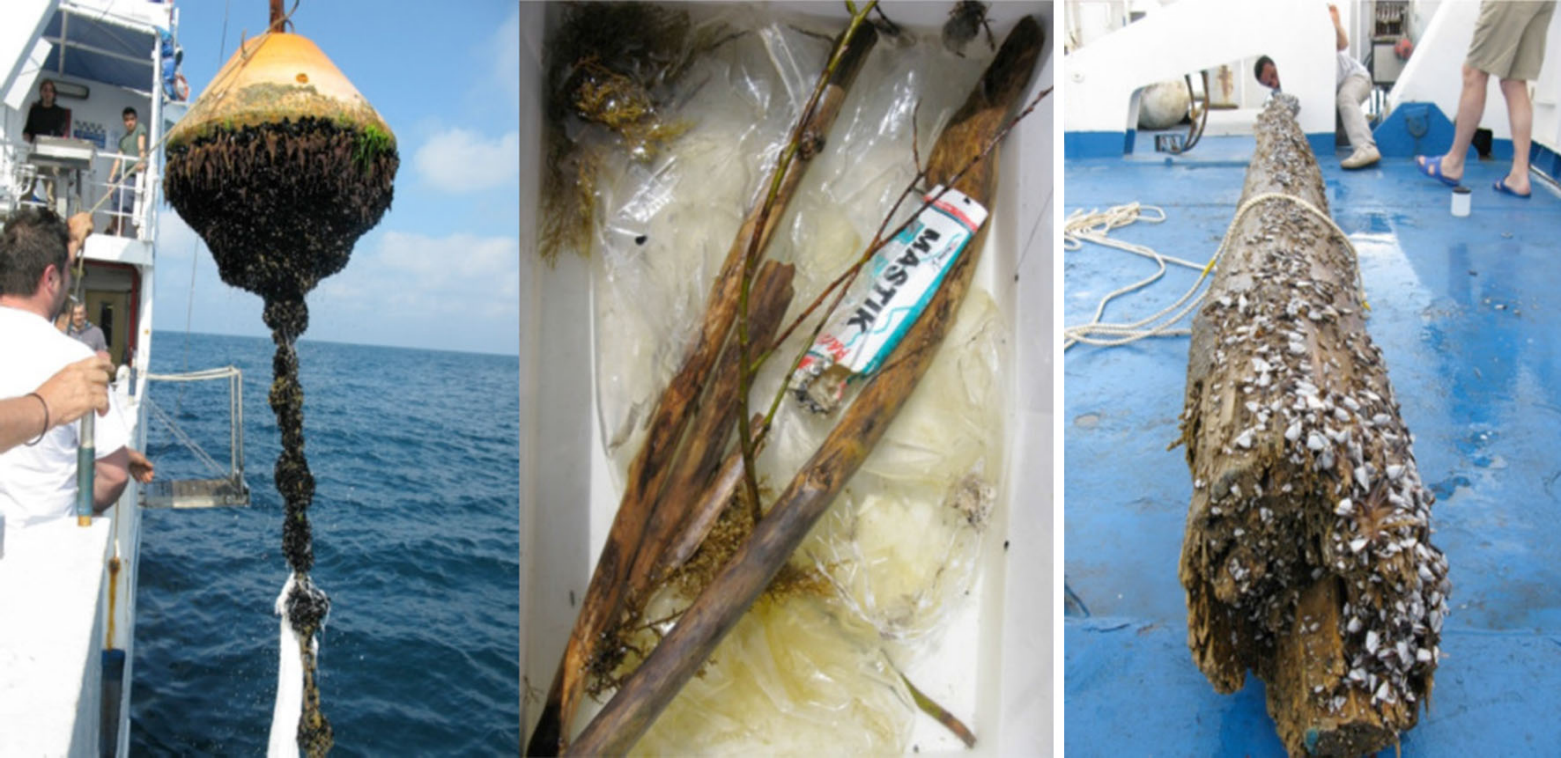
Photos of floating marine debris sampled in Southern Adriatic during CoCoPro Cruise in 2012. Many adult specimens potentially able to reproduce on flotsams are visible in the photos

Hydroids, molluscs (gastropods and bivalves), polychaetes (mainly Serpulids and Nereids), and crustaceans (Lepads, Amphipods, Isopods and Decapods) were found living on the objects collected. Some of these species, as the gooseneck barnacle *Lepas anatifera* and the associated nudibranch *Fiona pinnata*, are super wanderers and are usually found in offshore fouling. However many coastal species were also found. In particular, 1–2 cm *Mytilus* sp. specimen completely covered large objects, such as detached mooring buoys, and entire miniature communities (including the shore crab *Pachigrapsus marmoratus* and several species of benthic nudibranchs) were found living on the drifting mussel beds. It has to be noted that this type of transport of portion of benthic communities supplements the transport of pelagic larvae in ways that are not accounted for yet. An example of these hitchhiking communities on rafting objects is shown in Fig. 4.

### GUARD1: automatic visual detection system for planktonic species

**Fig. 5:**
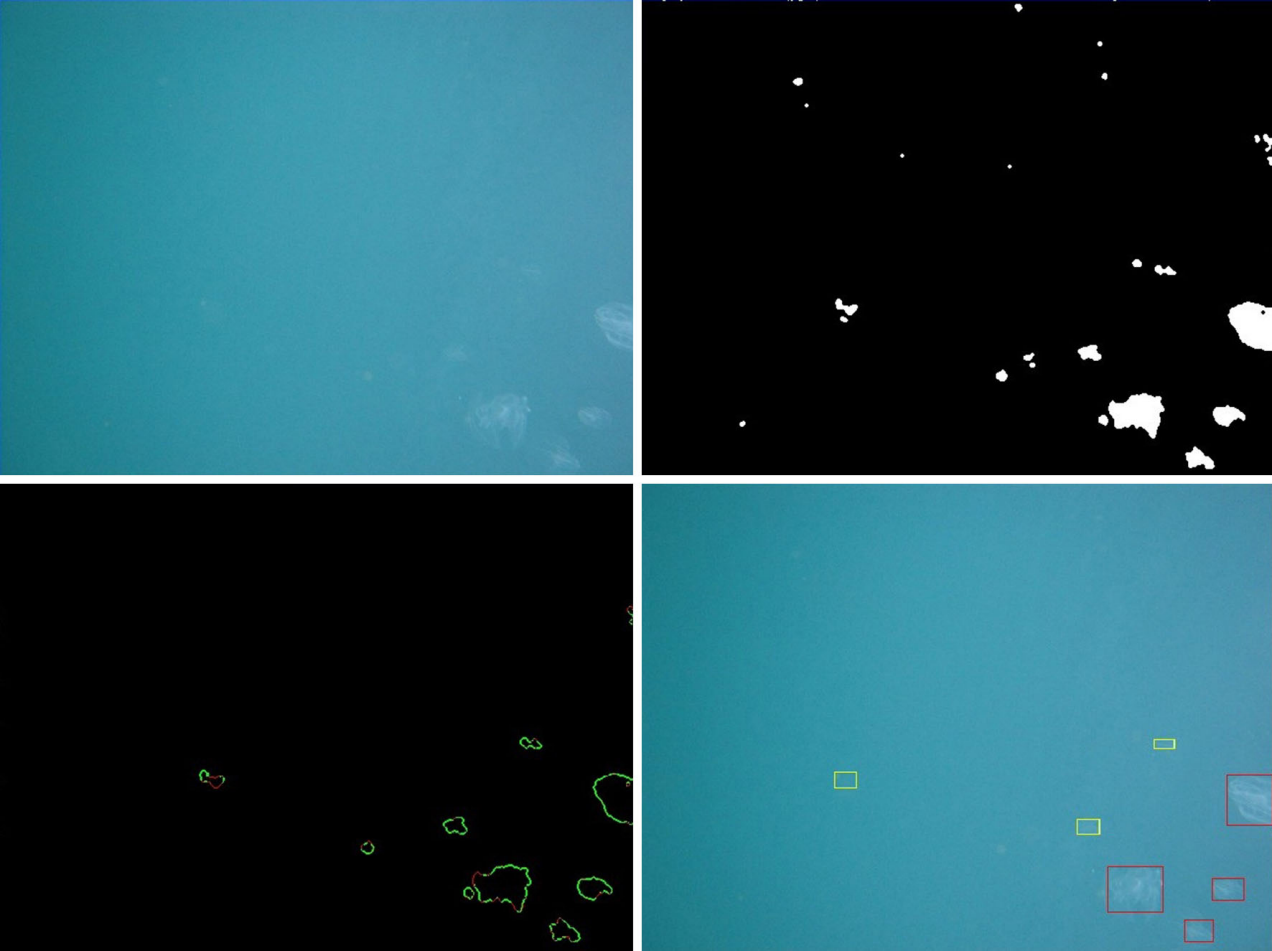
Image processing steps: original image where the specimens are almost invisible due to their transparent body (*top left*), binarized image (*top right*), contours of the relevant objects (*bottom left*), automatic detection on the original image (*bottom right*)

Another approach towards understanding planktonic dispersal is to directly monitor plankton. To this aim, a novel, autonomous imaging device (GUARD1) is currently being developed (Marini et al. 2013). The system has onboard automatic image elaboration capability for the recognition of gelatinous zooplankton and it is designed to autonomously operate on both fixed and mobile platforms. GUARD1 has been so far tested in several experiments led in controlled environment, at the Aquarium of Genova (Italy), and in the Ligurian and the Tyrrhenian Sea between 2012 and 2014. The experiments have been performed in order to validate the imaging and recognition performances of zooplankton in the context of the coastal scenario. Figure 5 shows the image processing and feature extraction pipeline from a sample image. The camera-collected image is reported in the left panel, its binarized version is in the center left panel, the contours of the relevant foreground objects are in the center right panel and the detected regions of interest (blobs) are in the right panel. The recognition module was able to automatically identify, through its interpretation pipeline, both gelatinous zooplankton and other marine instances of potential interest and was able to successfully detect two blooms of the ctenophore *Mnemiopsis leidyi* that took place in 2014 in the Ligurian Sea area. We plan to use it in the future in the Adriatic to capture images of other zooplankton groups like copepods, as well as marine litter along the dispersal paths starting from MPAs along the Italian coast.

**Fig. 6:**
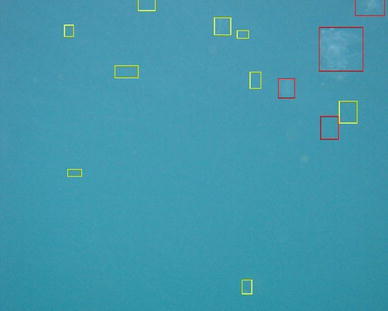
GUARD1 device automatically identified ctenophores during a bloom in the Ligurian Sea. An example of automatic acquired image and recognition of patterns therein is reported. The *darker boxes* highlight ctenophores and the *brighter boxes* highlight other foreground instances

An example of automatic detection for ctenophores is reported in Fig. 6. The recognition module provided high detection rate and good generalization capability (Corgnati et al. 2014), achieving an accuracy of 85.9 % in detection of gelatinous zooplankton specimen, with a true positive rate of 84.6 %, a false positive rate of 11.6 % and a false negative rate of 15.4 %. Sorting a small and robust set of relevant features in a large amount of images is crucial for avoiding computational consuming pre-processing and energy consumption. These are basic requirements for autonomous long-lasting imaging systems.

## Discussion and conclusions

As stressed by several authors (Barry and Dayton 1991; Mann and Lazier 2009), it is the existence of physical factors acting together with biotic relationships that controls the distribution of organisms. Nonetheless, although it is widely accepted that the contribution of dispersal patterns in the water column is a source of potential variability (Boero 1994), experimental studies concerning the coupling of physical and biological processes are still in their infancy. In this work, we present different approaches towards understanding natural and anthropogenically enhanced dispersion processes, with the ultimate aim of contributing to a better knowledge of connectivity between MPAs.

Radar data and numerical models provided information on the hydrodynamical processes in the Gulf of Manfredonia and Gargano area, with consequences on processes of dispersion and retention. Wind is found to be a major force affecting circulation patterns and offshore transport.

The general circulation features of the Gargano area were first described during the DART experiment (Haza et al. 2007), which showed a main boundary current flowing southward along the Italian coast. In the interior of the Gulf, a large anticyclonic recirculation is generated by the process of separation behind a cape (Magaldi et al. 2008). Dispersal paths of species are expected to follow this general pattern. Of particular interest are the filaments directed offshore which are expected to enhance cross-shelf exchanges. These processes may be important for the natural, long distance dispersal of coastal species (Shanks 1986), as coastal propagules are not only retained locally but can interact with the general offshore circulation patterns of the Adriatic.

In practical terms, if we want to model the evolution of a cloud of dispersal propagules, we face the problem that uncertainty in prediction quickly increases in time because of mortality (e.g., predation or length of life cycle), diffusion and advection. By using the LAVA blending technology to estimate Lagrangian transport, the uncertainty on particle position shown by model and radar velocity fields is reduced from 14–15 km to about 1–2 km over 24 h for both radar and model applications. LAVA is therefore a useful tool to increase tracking capabilities, especially if *ad hoc* releases of drifters are performed in the area at the time of interest.

We also should consider that measuring MPAs connectivity should take into account that marine ecosystems are affected by climate change at multiple levels, e.g., current dynamics and dispersal as well as species physiology and species densities. We have described conditions with cross-shelf filaments and along-coast currents with local recirculation that are occurring under present day forcing conditions. Proper measuring technique, in addition to integrating disciplines, as in the shown example, will be able to face future scenarios due to climate change. Changes in climate patterns alter sea currents and water transport, which in turn modify the hydrographic properties and nutrient content of the water column, which can result in large scale changes in the marine community (Conversi et al. 2010; Greene et al. 2013). The ongoing warming on the other hand is shifting the biogeographical range, usually northwards (Beaugrand 2012; Beaugrand et al. 2014), is altering the phenological cycles of many species (Mackas et al. 2012) and introducing new dispersal pathways and means.

Propagules are the actors of dispersal. Many dispersal forms have been identified ranging from larvae, to cysts, to living adults, to parasites of large animals. Collection of samples is the traditional method to identify organisms in the water column. However, such collection usually follows a fixed, grid/transect (Eulerian) sampling scheme, which, although very good for biomass/abundance estimates of marine organisms, is not appropriate for following propagule dispersion. A Lagrangian sampling scheme, which follows the water current, is more appropriate for dispersion studies. The use of unmanned, low-cost, high-deployment-range mobile image acquisition platforms, as the GUARD1 system presented here, can therefore fill this need, because they can be included in drifters, profiling floats or gliders to document biological or litter transport.

Man-made objects are now more abundant than any other natural floating substrata in most of the world’s oceans, including the Mediterranean Sea (Suaria and Aliani 2014), and they provide additional means of transport along and across water masses. To date, over 270 marine species have been reported associated with floating debris, with the most commonly found organisms including barnacles, polychaetes, bryozoans, hydroids and molluscs (Aliani and Molcard 2003; Barnes and Fraser 2003; Barnes and Milner 2005; CBD 2012; Goldstein et al. 2014). In the Adriatic Sea, the vast majority of all floating items are now of anthropogenic origin, a large number of which are intensively fouled by marine fauna. As a result, since the introduction of man-made litter into the marine environment, the possibility for organisms to find objects on which to attach has greatly increased. However, the implications that such an increase may have for the connectivity of the entire Adriatic basin are not yet known and should be further investigated in more detail.

In conclusion, the description of dispersal processes via the multidisciplinary approach presented in this paper provides complementary angles of view. Tools exist to better predict and describe dispersal processes that integrate data acquisition, models and new softwares for accurate data analysis to be coupled with ecological and biological information acquired automatically or traditionally.

- Studies on the dynamics of water currents indicate that the Gargano area has the potential to supply dispersal propagule both to the Southern Adriatic along the Italian coastline and offshore across the basin. Thus the nearby MPA of Tremiti Islands is expected to provide important service to the dispersal processes that sustain biodiversity of the Southern Adriatic.
- Softwares for data analysis, as LAVA, can help to predict natural dispersal paths more accurately than before and it can be used in an on-demand scenario to describe dispersal properties of the water column.
- New technology has been developed that automatically identify spatial or temporal distributions of selected species in the water column via image analysis. The system has so far successfully detected very active predators living in the water column, such as ctenophores, and is currently being evaluated for other types of identification, such as litter or marine copepods.
- Potential dispersal opportunities by rafting of living adults/juveniles has increased by an almost 4-fold in the central-southern Adriatic Sea, due to the introduction into the marine environment of artificial floating objects. Dispersal pathways can be predicted by current measurements and models, but the ecology therein is poorly studied. Studies of community dynamics on rafts are necessary to properly understand the real ecological potential that an increase in rafting opportunities can have for Mediterranean ecosystems.

A multidisciplinary perspective is crucial in order to provide a new ecosystem-based vision to the managers in charge of the marine environment and MPAs.
